# Experiences of Family Members and Patients with Spinal Muscular Atrophy Under the Multi-Level Medical Security System in Shaanxi Province, China: A Mixed Study

**DOI:** 10.3390/healthcare13020140

**Published:** 2025-01-13

**Authors:** Yuhan Zhao, Shengjie Ding, Chenglong Lin, Yubei Han, Mingyue Zhao

**Affiliations:** 1 Department of Pharmacy Administration and Clinical Pharmacy, School of Pharmacy, Xi’an Jiaotong University, 76 Yanta West Road, Xi’an 710061, China; zyh010918@163.com (Y.Z.); shengjie_1999@163.com (S.D.); cpulinchenglong@163.com (C.L.); ybhan2000@163.com (Y.H.); 2 Center for Drug Safety and Policy Research, Xi’an Jiaotong University, Xi’an 710061, China

**Keywords:** spinal muscular atrophy, qualitative research, multi-level medical security system, self-perceived burden

## Abstract

**Introduction:** Spinal muscular atrophy is a rare genetic disease. Nusinersen and Risdiplam, recognized as disease-modifying therapies, were included in the National Reimbursement Drug List in 2022 and 2023, respectively, in China. Policies have been implemented to enhance a multi-level medical security system, particularly for rare diseases. This study explores the self-perceived burden and offers policy suggestions to improve China’s social security for rare diseases. **Methods:** In our mixed study, we conducted 37 semi-structured online interviews and a quantitative survey with 3 adult SMA patients and 34 family members (primary caregivers) in collaboration with the Meier Advocacy and Support Center. The interviews explored self-perceived burdens in psychology, domestic relations, medical care, rehabilitation, and economy, analyzing mainly through thematic analysis and multiple linear regression. **Results:** Respondents reported significant psychological burdens mainly stemming from limited treatment access. The instability within these families was linked to inconsistent therapeutic schedules, the lack of development opportunities, and misunderstandings. Choices between institutional and home rehabilitation were influenced by economic conditions and symptom severity. After the inclusion of medications, six patients (16.2%) still had not received pharmacological treatment, and many of those who underwent treatment were dissatisfied with the outcomes. The high costs of rehabilitation, family labor loss, and an incomplete medical security system resulted in significant economic burdens. Respondents called for more effective medications and better patient support. **Conclusion:** Although the inclusion of medications in National Reimbursement Drug List has improved availability and affordability, families still experienced significant burdens across multiple domains. A broader focus on social security is needed to enhance the comprehensive development of patients with rare diseases.

## 1. Introduction

Spinal muscular atrophy (SMA) is an inherited neuromuscular disease characterized by the degeneration of spinal cord motor neurons, leading to skeletal muscle atrophy and generalized muscle atrophy [1,2,3]. The clinical manifestations include progressive, symmetrical proximal limb muscle weakness and premature death [4]. Based on the age of onset and clinical phenotype, SMA is classified into types I to IV [5]. The estimated prevalence is approximately 1 to 2 per 100,000 people, with SMA type I, the most severe form, accounting for about 60% of all cases [6,7].

Initially, the management of SMA was limited to symptomatic therapies, including respiratory support, nutritional support, and rehabilitation support [8]. Subsequently, disease-modifying therapy referred to interventions that affect the pathophysiological processes of a disease to produce beneficial outcomes [9]. Thereafter, gene replacement therapy was developed to target the genetic cause of spinal muscular atrophy [10]. In 2016, the FDA approved Nusinersen for the treatment of SMA in both children and adults [11]. Four years later, Risdiplam became the first oral small-molecule treatment available [12]. Both therapies could improve the symptoms of the patient’s disease [13] and modify the disease progression [8]. In 2019, the FDA approved Zolgensma, an innovative gene therapy administered as a single intravenous infusion with systemic availability, for treating pediatric patients [14,15]. In China, Nusinersen and Risdiplam were included in China’s National Reimbursement Drug List (NRDL) in 2022 and 2023, respectively [16,17]. Through national medical insurance negotiation, the price of Nusinersen was reduced from the original CNY 699,700 per injection to CNY 33,000 per injection, representing a reduction of approximately 95.5%. Risdiplam was initially priced at CNY 63,800 per bottle, but following negotiations in 2022, the price was decreased to CNY 3780 per bottle, reflecting a reduction of about 94.1%. To date, Zolgensma has been approved for marketing in the United States [18], the European Union [19], and other countries, while in China, it has only received approval for clinical trials.

China’s policy framework constantly enhances the construction of a multi-level medical security system, particularly regarding the protection of rare disease populations. In 2020, the Central Committee of the Communist Party of China and the State Council jointly issued the “Opinions on Deepening the Reform of the Medical Security System”. This policy [20] mandated the establishment of a comprehensive system in three tiers: the first tier consisted of the government-led basic medical insurance layer, which included basic medical insurance, critical illness insurance, medical assistance, and long-term care insurance. The NRDL set the standard for reimbursing drug costs under basic medical insurance, while major illness insurance covered high costs from serious diseases, and medical assistance provided subsidies for low-income families. Additionally, long-term care insurance supports individuals disabled by illness, alleviating financial burdens and collectively safeguarding health while preventing illness-induced poverty. The second tier was the commercial insurance layer, provided by insurance companies, which offered personalized and diverse options for more comprehensive healthcare coverage. The third tier was the social assistance layer, which included charitable aid, social assistance, social welfare, and preferential treatment and resettlement, aimed at providing essential support to the most economically disadvantaged populations and improving healthcare accessibility. This macro policy offered guidance for crafting rare disease policies [21], emphasizing the critical role of the medical security system in enhancing drug availability and healthcare quality. However, China’s multi-level medical security system remains in its early stages, necessitating further enhancements to fully support patients with rare diseases [22].

Previous studies primarily examined patients’ experiences, psychological impacts, caregiver burdens, and social support. Two qualitative studies [1,3], one from the United States and another from Spain, highlighted key challenges for SMA patients, including isolation, stigma, and loss of independence. Another study [23], employing semi-structured interviews, identified mental health care as a critical unmet need for SMA patients but noted resilience with support from family and peers. Caregivers reported feelings of uncertainty and powerlessness in managing care [24]. Additionally, an Australian study [25] emphasized the importance of connecting with SMA-experienced communities to alleviate loneliness and improve access to treatment-related information. However, there is currently a lack of research on the self-perceived burden (SPB) among family members and patients with SMA in mainland China, highlighting the necessity for further research.

Our goal is to investigate the SPB experienced by family members and patients with SMA in terms of psychology, domestic relations, medical care, rehabilitation, and economy under the multi-level medical security system. We focus on the changes before and after the implementation of the policy, primarily utilizing semi-structured interviews and quantitative surveys. We aim to propose policy recommendations based on the findings of this study, with the goal of continuously enhancing the development of China’s social security network for rare diseases.

## 2. Materials and Methods

### 2.1. Study Design

This mixed study used both qualitative and quantitative research methods to evaluate the burden of family members and patients with SMA under the multi-level medical security system. Through a review of the published literature, we initially formulated five key aspects for the interview, including psychology, domestic relations, medical care and rehabilitation, economy, and appeals. Following expert opinions, we revised and refined the interview guide. Furthermore, the exact wording of the questions and the order of responses were left open. In terms of psychological aspects, participants were asked to complete the Depression–Anxiety–Stress Scale (DASS-21) to conduct a quantitative study of their current psychological burden [26]. Also, we employed the Likert Scale to assess the satisfaction with the effectiveness of both medication and rehabilitation treatments. Two experienced interviewers conducted face-to-face pre-interviews with a patient’s mother, one of whom served as the main interviewer and the other as a supplementary interviewer and recorder. Based on the feedback from the pre-interview, we adjusted the content of the interview accordingly. First, we removed questions on clinical indicators, such as the Children’s Hospital of Philadelphia Infant Test of Neuromuscular Disorders, as respondents could not clearly recall the results, which were typically retained in hospital records. Second, to address privacy concerns, we gently broached the topic of marital status by initially inquiring about family size and permanent household members. Additionally, we determined that the follow-up interviews would be conducted online and scheduled to last between 30 and 60 min.

### 2.2. Participant Recruitment

Participants were included in this study if they were family members of SMA patients or adult patients who were knowledgeable about their own treatment and costs, demonstrated good compliance, and resided in or received treatment in Shaanxi Province, China. Participants were excluded if they or their family members had a disturbance of consciousness or were currently enrolled in a clinical trial. Given the low prevalence of SMA as a rare disease, participants were recruited through the Meier Advocacy and Support Center, the only professional and authoritative nonprofit organization specializing in SMA in China. We contacted the principal of the Meier Advocacy and Support Center in Shaanxi Province via email, and we posted a recruitment notice on the WeChat groups, outlining the study’s purpose and significance. Through voluntary sign-up and principal recommendations, 42 families were invited. However, 5 families withdrew before the interviews due to privacy concerns regarding the recording requirement. Therefore, we obtained a total of 37 valid responses. To assess the reliability of the sample size, we obtained data from the Shaanxi Provincial Medical Insurance Database, which reported 34 patients using Nusinersen in 2022, closely aligning with the 27 patients in 2022 in our study. Additionally, our study included patients not receiving pharmacological (n = 6) or rehabilitation (n = 5) treatments, thereby enhancing sample diversity and the generalizability of the findings. Among the 37 respondents, 3 were adult patients, and the remaining 34 were family members (primary caregivers). All respondents provided verbal informed consent and received compensation based on the interview duration.

### 2.3. Data Collection

We used semi-structured interviews to conduct in-depth interviews. Our interviews focused on two main aspects. The first aspect explored whether family members and patients experience changes in psychology, domestic relations, medical care, rehabilitation, and economy within the multi-level medical security system in China, considering the increased availability and affordability of medical resources. The second aspect focused on the urgent appeals. In quantitative terms, each respondent completed a basic demographic survey, a DASS-21 questionnaire, and self-assessed satisfaction regarding medication and rehabilitation treatments. Two authors conducted and recorded the study online in October 2024.

### 2.4. Data Analysis

#### 2.4.1. Qualitative Data Analysis

This study used thematic analysis [27]. Two analysts reviewed the transcripts and standardized the text by removing colloquialisms and repetitive statements from the narratives. During this process, all identifying information was anonymized, and respondents were assigned numbers. In the initial phase, open coding was used to analyze the transcripts. One analyst assigned preliminary themes and sub-themes to text segments relevant to the research questions. Two analysts independently reviewed the transcripts, coded the data, and refined the emerging themes and sub-themes. They subsequently compared their findings, engaging in a discussion to reconcile similarities and discrepancies. Core concepts, related experiences, and interconnections were recorded for each theme. In the second phase, when open codes involved similar themes (e.g., whether improvements in physical mobility were attributed to psychology or medical care), the third analyst reviewed the data content. Through in-depth discussion and negotiation, the three analysts reached a consensus on resolving our differences, achieving over 90% agreement, and reclassifying the ambiguous codes. In the third phase, the relevance of each theme to the research questions was evaluated, and hypotheses were constructed to examine the relationships between the themes.

#### 2.4.2. Quantitative Data Analysis

We conducted double data entry using EpiData.3.1 Following data quality control and cleaning, we performed descriptive statistical analyses of subgroups using Excel 2022 and further statistical analyses with Stata 17.0. The DASS-21 scale was used to assess scores across various dimensions, and multiple linear regression analysis was conducted to explore the correlations between these scores and psychological indicators based on predefined classification criteria. Depression was selected as the primary dependent variable, and several independent variables were initially identified based on baseline data and semi-structured interview results. Given the small sample size (n = 37), six variables were ultimately included to achieve optimal model fit: type of SMA, family income, participation in civil relief, whether full-time caregiver, the number of caregivers, and disability level. And we described the results of self-assessment of medication and rehabilitation satisfaction using the Likert five-point scale, with respondents’ answers categorized as “very dissatisfied”, “dissatisfied”, “neutral”, “satisfied”, and “very satisfied”.

### 2.5. Ethics

The study received approval from the Research Ethics Committee of The First Affiliated Hospital of Xi’an Jiaotong University (XJTU1AF2024LSYY-218). Informed consent was obtained from all participants. We ensured the confidentiality and privacy of participants throughout the study. Any identifiable information was anonymized during data analysis and reporting.

## 3. Results

### 3.1. Study Sample

We described the demographic and clinical characteristics in terms of patient details, current disease state, and caregiver and family information. A total of 37 respondents participated, with online interviews conducted with three adults and the remaining interviews conducted with their family members (primary caregivers). The ages of SMA patients ranged from 1 to 40 years. The mean age of all patients was 10.0 (7.0) years. The majority of patients were diagnosed with type II SMA (56.8%), followed by type III SMA (29.7%), while four patients had type I SMA (10.8%), and one patient had type IV SMA (2.7%). All patients had purchased urban and rural resident basic medical insurance. Among them, 81.1% had purchased commercial insurance, and 75.7% had inclusive commercial insurance. Among the respondents, 62.2% reported an annual family income of less than CNY 60,000, 24.3% had an income of 70,000 to CNY 90,000, 10.8% had an income of 100,000 to CNY 130,000, and only one family had an income of more than CNY 220,000. The results are presented in Table 1.

### 3.2. Psychology

Family members and patients expressed deep despair and hopelessness upon the first SMA diagnosis, especially given the lack of treatment options. Some struggled to accept the diagnosis, leading to disbelief that their family could be affected by such a rare condition. One parent said, “*After our child was diagnosed with SMA, we felt hopeless and experienced sleepless nights searching for information*” (Patient-5, Type II). Because of the extremely high burden in life, the respondent even had a radical idea: “*The lack of hope led to profound despair, and we felt so guilty about the genetic disease that we even thought about suicide with our child*” (Patient-30, Type II). During the period when Nusinersen was available in the U.S. but not in China, parents felt a heightened sense of helplessness: “*It was agonizing to know the drug was out there but inaccessible to my child*”, said one mother (Patient-16, Type II).

Following the inclusion of SMA medications in the NRDL, families expressed newfound hope: “*The policy gave us hope that our child might walk within a couple of years*”, said one parent (Patient-6, Type II). Simultaneously, a few parents (n = 3) relied on feedback from patients who had already taken medication, leading them to doubt whether these drugs could produce effective therapeutic results: “*My child has severe scoliosis, so I concern that the treatment might cause him significant pain*” (Patient-34, Type II).

After pharmacological treatment, a small number of respondents (n = 7) were satisfied with the therapeutic outcomes, attributing this to observed improvements in their children’s daily mobility and the affordability of the medication within their financial means. However, conversely, most respondents expressed deep disappointment with the outcomes, citing increasingly significant family conflicts and economic pressures stemming from the disease.

We used the DASS-21 scale to measure the psychological stress and influencing factors among family members and patients in our study. The questionnaire survey regarding the caregivers’ basic situations revealed that respondents continued to experience a significant psychological burden. The annual household income and role of being the primary caregiver emerged as key factors contributing to stress, anxiety, and depression. The results are summarized in Table 2.

The results of multiple linear regression revealed that family income (β = −0.70, *p* = 0.001) and participation in civil relief (β = −1.21, *p* = 0.004) had significant negative predictive effects on depression levels, indicating a potential protective role of these factors against depression in SMA families. Higher family income may alleviate the financial strain associated with the intensive medical and rehabilitation required for SMA management, thereby reducing depression among family members. Similarly, participation in civil relief programs, such as receiving a subsistence allowance, can help offset the high costs of disease, contributing to a lower risk of depressive symptoms. Conversely, whether full-time caregiver (β = 1.41, *p* = 0.005) and the number of caregivers (β = 0.48, *p* = 0.009) showed significant positive predictive effects, suggesting these factors may exacerbate depression. Full-time caregiving responsibilities can lead to physical and emotional exhaustion, social isolation, and financial strain, significantly increasing depression levels. Similarly, a higher number of caregivers may indicate greater disease severity or care complexity, which in turn also increases the psychological burden on caregivers. Additionally, the type of SMA (β = 0.55, *p* = 0.055) was borderline significant, implying a potential association with depression severity, while the impact of disability level (β = 0.33, *p* = 0.061) was not statistically significant. The overall model demonstrated statistical significance (F = 6.19, *p* = 0.0003) with an adjusted R^2^ of 0.4636, indicating that the independent variables explained approximately 46.4% of the variance in the dependent variable. These findings highlight that family income, caregiving burden, and participation in civil relief are critical factors influencing depression levels, providing valuable insights for the implementation of targeted intervention strategies in the future.

### 3.3. Domestic Relation

In our study, all families stated they were married before the SMA diagnosis. Post-diagnosis, some families stayed united in facing the disease’s challenges: “*We’ve agreed on our child’s treatment, and our relationship is strong*”, said one parent (Patient-35, Type II).

However, relationships between other families altered because of the therapeutic schedule and changes in family responsibilities. Among them, most family (n = 10) relationships became unstable. The inconsistent therapeutic schedule was a key issue, as one mother explained, “*The father and I disagreed on the treatment—he thought it was unnecessary and costly, while I wanted to continue*” (Patient-4, Type III). The need for one parent to give up further education or work to care for the child also strained relationships. “*I had to quit my well-paying job to care for our child, leading to frequent conflicts with my husband*” (Patient-34, Type II). Misunderstandings about SMA among family members caused significant conflicts: “*Due to the poor education level, my father-in-law and mother-in-law initially thought it was my problem caused the child’s illness, and there was a big conflict between us*” (Patient-36, Type II).

Four families ended in divorce over SMA-related disputes, with mothers taking sole charge of raising the children. One mother recounted, “*Discussing our child’s treatment with her father led to a major argument, and we divorced, drastically changing my family structure*” (Patient-7, Type I).

### 3.4. Medical Care and Rehabilitation

Prior to the inclusion of drugs for SMA in the NRDL, treatment mainly relied on rehabilitation, which was categorized into institutional rehabilitation (hospital or professional institutions) and home rehabilitation. Due to the high expenses, only a subset of (n = 10) patients opted for institutional rehabilitation: “*We have professional rehabilitation in the hospital five times a week, but at the same time the costs are significant and the travel distance is considerable*” (Patient-31, Type II).

Home rehabilitation offered a more affordable and convenient alternative to institutional rehabilitation. However, without professional guidance, families struggled to provide effective rehabilitation and nursing care, leading to suboptimal rehabilitation outcomes for patients. One mother reported: “*Initially, we didn’t know how to do rehabilitation exercises, so we just followed online videos to help him at home*” (Patient-21, Type III). Additionally, the lack of patient initiative meant constant family support was needed, as another parent explained, “*Home rehabilitation required our full-time care and attention*” (Patient-14, Type III).

However, some families (n = 5) gave up rehabilitation treatment because of severe symptoms and loss of limb movement ability: “*Given my child’s young age and limited ability to move, as he could only move his eyes, we opted not to pursue rehabilitation treatment*” (Patient-1, Type I).

After the release of relevant policies on SMA, the demand for treatment significantly increased. Most patients received both rehabilitation and concomitant pharmacological treatment, with a total of 31 patients undergoing pharmacological treatment: 6 were treated with Nusinersen alone, 4 with Risdiplam alone, 19 with combination therapy, and 2 switched from Nusinersen to Risdiplam after initial treatment.

After pharmacological treatment, only seven respondents reported satisfaction. Caregivers noticed improvements in daily activities, “*Initially, my child was reluctant to go downstairs for a walk, but after starting the medication, I found that he could walk independently in the community with my encouragement*” (Patient-4, Type III). Another mother said: “*I am surprised to see my child fell significantly less after treatment*” (Patient-14, Type III).

Despite trying pharmacological therapy, either alone or in combination, most family members and patients did not see significant improvements. The high hopes initially held led to a considerable gap between expectations and reality, as expressed by one parent: “ *I had high expectations for the medication, firmly believing that it would allow her to lead a normal life. Yet, the reality was that there had been no improvement*” (Patient-24, Type II). Additionally, one of the mothers in our study mentioned: “*My child had severe scoliosis and it was very painful to use Nusinersen. Risdiplam also had the side effect of fever, so we stopped using drugs*” (Patient-34, Type II). Therefore, only 30 families are still under pharmacological treatment.

To our relief, society’s focus on SMA patients’ rehabilitation needs grew, with local disabled persons’ federations stepping in to provide professional services. “*My child attend hospital-based rehabilitation sessions in collaboration with the disabled persons’ federation monthly, which I believe aids recovery*”, shared one parent (Patient-20, Type II). Rehabilitation sessions also offered social interaction for patients: “*My child looked forward to rehabilitation at the Disabled Persons’ Federation for the chance to socialize, as the condition kept him staying at home*” (Patient-24, Type II). In addition to the Disabled Persons’ Federation, some social organizations were becoming involved in the rehabilitation and care of patients. Among these, the Meier Advocacy and Support Center stood out for providing online nursing and rehabilitation resources, as well as equipment such as wheelchairs and standing frames.

Respondents were asked to provide the family’s overall assessment and perception of the quality and effectiveness of medical and rehabilitation care. The self-assessment of medication and rehabilitation satisfaction is presented in Table 3. Regarding disease classification, 3 respondents (75.00%) with type I SMA and 14 respondents (93.33%) with type II SMA reported that the treatment effect was dissatisfied or general. Two respondents (66.67%) and thirteen respondents (72.22%) thought that the rehabilitation effect was dissatisfied or general. However, among patients with type III SMA, six respondents (54.55%) considered the treatment effect to be dissatisfied or general, and seven respondents (70.00%) considered the treatment effect to be dissatisfied or general. Among the patients without scoliosis, seven respondents (77.78%) thought that the treatment effect was dissatisfied or general, and five respondents (55.56%) thought that the rehabilitation effect was dissatisfied or general. Among the patients with scoliosis, 17 respondents (77.27%) thought that the treatment effect was dissatisfied or general, and 18 respondents (78.26%) thought that the rehabilitation effect was dissatisfied or general.

### 3.5. Economy

Family members and patients endured substantial economic burden. Prior to the inclusion of Nusinersen and Risdiplam in the NRDL, only two families in our study could barely afford the costly drugs. “*We sold a house and spent over a million on our child’s treatment in 2021*”, said one mother (Patient-37, Type II). Another noted, “*We got assistance from Roche and the China Women’s Development Foundation in 2021, paying CNY 190,000 for three bottles of medication and getting six more free. But the costs were too high, so we switched to Nusinersen after it was covered by the NRDL in 2022*” (Patient-26, Type III).

However, the economic burden for the families of other patients primarily stemmed from the costs associated with rehabilitation and medical equipment. A few wealthier families (n = 3) enrolled their children in one-on-one rehabilitation courses, including rehabilitation sports and swimming lessons: “*One-on-one lessons are expensive but necessary for his rare condition*” (Patient-4, Type III). Others spent on basic hospital rehabilitation: “*We’ve been doing muscle exercises since diagnosis, which is costly for us*” (Patient-10, Type II). Some families opted for home-based rehabilitation: “*It requires one of us to help her with exercises to maintain her mobility; however, some equipment is quite expensive*” (Patient-13, Type III).

SMA patients’ limited mobility necessitated full-time caregivers, leading to a loss of family labor. One father described the financial strain: “*With my father’s bladder cancer, my daughter’s leukemia, and now my son’s SMA, I’m the sole breadwinner*” (Patient-18, Type II).

Following the inclusion of Nusinersen and Risdiplam in NRDL, with the exception of the two patients already receiving treatment, most of the remaining families (n = 29) gained hope for effective therapy and turned to medication alongside rehabilitation. In 2022, in addition to the aforementioned reasons, the substantial cost of medications imposed an increased economic burden, shifting the main expense from rehabilitation to medication costs. One mother of the SMA patient recommended: “*I’m relieved there’s a treatment for SMA, yet the cost is hard to bear due to the need for lifelong treatment*” (Patient-27, Type II). The expense deterred six families from using medication: “*We want to start medication but can’t afford it on our low income*” (Patient-17, Type II).

Despite the increasing national attention to rare diseases, the multi-level medical security system remained incomplete. Basic medical insurance was meant to provide essential medical care, but policy implementation faced challenges. In the reimbursement process, hospitalization was mandatory: “*Outpatient treatments for drugs weren’t reimbursable; only inpatient care qualified. The process was difficult and required tests and bloodwork, which the child found very painful*” (Patient-15, Type II). The reimbursement rate was also a concern. Several respondents found the coverage inadequate: “*Basic medical insurance offered limited coverage for this disease. Without subsistence allowances, a Nusinersen injection cost about CNY 12,000, and for most families, a 50–60% reimbursement was insufficient*” (Patient-2, Type III). Disparities in rates between provinces created further challenges: “*We’re not registered in Xi’an but get treated here, so our insurance rates aren’t the same as local children’s*” (Patient-4, Type III).

During the interviews, many respondents mentioned trying to buy supplementary commercial insurance. However, as a genetic disease, most SMA patients were unable to obtain certification. One mother pointed out: “*Insurance applications were complex*” (Patient-12, Type II). Of the few who successfully acquired supplementary commercial insurance, they found the deductibles excessive and claimed the process was difficult: “*Lowering the minimum payment would really help us*” (Patient-8, Type I).

Medical assistance complemented the multi-level medical security system, yet its awareness was low. Many individuals in remote areas had limited awareness of it: “*We weren’t aware of the policy and hadn’t been informed that we could apply*” (Patient-9, Type II). Medical assistance also faced challenges in certification: “*I’ve tried multiple times to get medical assistance from the Civil Affairs Bureau without success*” (Patient-10, Type II).

Fortunately, the government and society have increasingly focused on patients with rare diseases. Some social organizations have joined in to help patients with rare diseases, offering some relief through donations of medical equipment. However, the capacity of social assistance remains limited, and it cannot directly address patients’ medical expenses.

### 3.6. Appeal

Family members and patients expressed concerns regarding quality of life, economic burden, and psychological pressure. In our interviews, they mentioned that the biggest appeal at present was mainly for new drug marketing and patient personal development. Firstly, as current pharmacological treatment had not met families’ expectations, nearly half of the respondents (n = 16) expressed hope for the availability of new drugs with improved efficacy to replace existing treatments. One father stated: “*We hope for more effective and prominent drugs*” (Patient-23, Type III).

Additionally, the second public appeal of the respondents was the patient’s personal development, including education and employment opportunities. “*My son wrestles in his daily life; though he’s school-aged, no school accepts him. I wish government could solve this problem*” (Patient-14, Type III). One adult patient shared: “*Our family income currently relies on my parents, and I can only do some part-time work. I also hope to gain full-time employment to alleviate my family’s economic burden*” (Patient-19, Type IV).

## 4. Discussion

To our knowledge, this is the first study to use Shaanxi Province in China as a case study, focusing on SMA as an entry point for understanding rare diseases in children. It examines the SPB on family members and patients in terms of psychology, domestic relations, medical care, rehabilitation, and economy. Respondents indicated that the disease imposed a significant burden across all these areas. Additionally, respondents reached a consensus regarding two main concerns: the marketing of new drugs and the personal development of patients.

Comparing our findings with previous studies, we find a consistent theme of the substantial psychological burden due to fear and disease uncertainty. However, the underlying reasons differ. In our study, parents were particularly concerned about treatment efficacy and their children’s survival. Based on the results of the DASS-21 scale, it can be concluded that economic and caregiving burdens have a significant impact on psychological depression. In contrast, an Australian study [28] suggested that parents positively shaped their child’s life and death after grieving, but they faced burdens, including losing future reproductive freedom. Furthermore, a previous study [1] reported a lack of peer companionship and feelings of being psychologically isolated. Nevertheless, the consensus from all current studies was that we should raise the psychological support [23,29,30].

Over one-third of the families in our study were in unstable family situations or divorced. However, the substantial negative effect of SMA on domestic relations was not evident in prior studies. According to a study from France [31], most parents were married and reported a good level of marital satisfaction, suggesting variations in marital protection policies could play a role. Further study is needed. Concurrently, other studies highlighted the significance of familial relationships in influencing treatment-related decision-making [25,32]. Our findings indicated that family decisions were influenced by the economic considerations of the disease’s financial and caregiving costs against potential benefits, with parents in stable relationships tending to make similar choices, while those in unstable relationships might make different decisions due to divergent value assessments.

Regarding the unreasonable expectations from pharmacological treatment, there were two potential causes: one was inadequate counseling by healthcare providers or media portrayal, and the other was that patients included in this study typically experienced disease onset prior to the availability and affordability of treatment options in China. As a disease-modifying therapy, medications failed to achieve the expected therapeutic effect.

Many countries are implementing policies to improve access to treatments for rare diseases like SMA. These include fast-track approval processes, risk-sharing agreements, and national insurance coverage to reduce financial burdens. Such initiatives aim to ensure equitable access to life-saving therapies for rare disease patients.

Australia has established a comprehensive rare disease policy, including an Expanded Access Program [33] that expedited regulatory reviews and reimbursements. Additionally, the inclusion of Nusinersen in the Pharmaceutical Benefits Scheme reduced patient costs significantly [34]. The National Strategic Action Plan for Rare Diseases, introduced in 2020, further bolstered care for SMA patients [35]. Therefore, a study from Australia reported that financial costs did not emerge as a factor when balancing the benefits and burdens of treatment [25].

Prior to the approval, Nusinersen was available to patients with type I SMA in Germany without restrictions on age groups and disease stages within the Expanded Access Program, boosting drug availability [36]. However, another study indicated that intensive contact with health insurance companies and repeated reimbursement make the economic burden heavy [24].

In 2015, the Dutch government implemented the Coverage Lock to evaluate new and high-cost medications [37]. However, a prior study showed that the Coverage Lock for Nusinersen took 15 months and imposed an age limit for reimbursement eligibility. The study also identified issues, such as the misuse of cost-effectiveness methods, which minimized Nusinersen’s impact on quality-adjusted life years [38]. Despite these concerns, the Netherlands provided reimbursement for Nusinersen for children under 9 years and 6 months, suggesting that medication costs are not a significant barrier to treatment access [39].

The American Orphan Drug Act was issued in 1983 to encourage pharmaceutical companies to develop drugs for the treatment of rare diseases. Despite vigorous state promotion of rare disease drug development, access to essential healthcare services remained challenging, as illustrated by Nusinersen, which was already covered by most commercial insurance [40]. It was difficult to obtain secondary insurance and access needed healthcare services [41].

South Korea has implemented comprehensive policies to support rare disease patients, focusing on reducing the high cost of treatments through the National Health Insurance Service and Risk-Sharing Agreements. Zolgensma, the treatment for SMA with a market price of about KRW 2 billion (USD 1.5 million), is covered by NHIS in South Korea, requiring patients to pay only KRW 5.98 million (around USD 4400) [33]. This policy significantly alleviates the financial burden on patients’ families.

The common appeal for better therapies was shared by our study and previous studies, including gene therapy [40], disease-modifying therapies [42], and healthcare advances [25]. Consistently, there was a shared focus on the personal growth of patients, including the child’s positive development and increased independence [43]. Furthermore, caregivers emphasized the necessity for specialized education or support services tailored for individuals [29].

This study has several limitations. Firstly, this study may be subject to selection bias for two reasons. The data was collected from SMA patients diagnosed or treated in Shaanxi Province, China, which may not reflect the national average due to regional differences in economic development and healthcare resource allocation. In the second place, reliance on voluntary participants could result in the overrepresentation of those with strong identification with the research topic, potentially affecting sample diversity and the generalizability of the findings. However, to the best of our knowledge, this is the first qualitative study in mainland China to evaluate the SPB of family members and patients from the perspective of a multi-level medical security system. Secondly, with the exception of three adult patients, caregiver reports dominated, potentially overlooking the direct experiences and concerns of the patients. Thirdly, the reliance on semi-structured interviews based on reporters’ memories could introduce recall bias. Finally, the majority of interviewees were mothers, which aligned with the trend that women assumed a larger caregiving role for individuals with disabilities [44].

This study has several implications. Firstly, based on the results of semi-structured interviews, we revealed the phenomenon that families of rare disease patients faced significant burdens. It aligned with recent findings on the health and economic impacts of rare diseases [45]. Secondly, the improvement and development of the multi-level medical security system require further strengthening. At the first level, beyond increasing medication reimbursement rates, national efforts should focus on enhancing professional care and rehabilitation for SMA patients. This includes strengthening the training and allocation of caregivers and rehabilitation specialists to ensure effective treatment, reduce caregiving burdens, and minimize workforce loss. The long-term care insurance system should be refined to cover rehabilitation and caregiving costs, alleviating the financial burden of illness and preventing poverty. Moreover, the government should prioritize the establishment of a comprehensive rare disease support framework, such as setting up a dedicated fund to guide policy implementation, enhance basic support for rare diseases, and thereby promote the joint efforts of all sectors of society in building a multi-level security system. At the second level, commercial insurance should develop special coverage for genetic diseases and enhance publicity efforts. It should align with national policies to provide supplementary economic coverage for rare disease patients. At the third level, social organizations should expand their role by enhancing community strength. To begin with, social assistance initiatives, such as expanding the “ice bucket challenge” and crowdfunding platforms like “waterdrop fundraising”, should be promoted to involve more charitable organizations and individuals in building a diverse rare disease support system. In addition, psychological education for patients and their families should be strengthened to alleviate familial and marital strain, promote positive communication, set realistic treatment expectations to reduce unnecessary stress, and, most importantly, help them understand what it’s like to live with SMA or have a child diagnosed with it. Furthermore, the multi-level medical security system should transform into an all-encompassing social security system, engaging diverse social forces to fulfill the therapeutic and developmental requirements of children with rare diseases. Future research should focus on children with normal cognitive abilities but physical disabilities, aiming to establish specialized educational pathways and facilitate access to higher education. This will ensure that their development aligns with societal needs, supports their employment, and maximizes their potential. Additionally, it is crucial to address the vulnerabilities of families affected by rare diseases, which are compounded by the challenges of the disease and economic hardship. Comprehensive support from various societal sectors is essential to facilitate their integration into the community, reduce life stressors, enhance quality of life, and create a more inclusive environment. These strategies are essential for promoting sustained well-being, social equity, and the full realization of the potential of individuals with rare diseases and their families.

## 5. Conclusions

In summary, this article revealed that under China’s multi-level medical security system, family members and patients with SMA experienced considerable SPB in terms of psychology, domestic relations, medical care, rehabilitation, and economy. Regarding the urgent appeal, respondents reached a strong consensus on the need for more effective drugs and greater attention to personal development. Future research should focus on transitioning from medical security to social security after enhancing the multi-level medical security system while also considering the multifaceted development of rare disease families.

## Figures and Tables

**Table 1:** Demographic and clinical characteristics of SMA study participants (n = 37 patient–caregiver pairs).

	SMA I	SMA II	SMA III	SMA IV	Total
Patient details					
Current age (mean (SD)), y	5.1 (4.8)	9.4 (3.9)	11.5 (10.1)	25.0 (0.0)	10.0 (7.0)
Sex, n (%)					
Male	2 (50.0%)	12 (57.1%)	5 (45.5%)	1 (100.0%)	20 (54.1%)
Female	2 (50.0%)	9 (42.9%)	6 (54.5%)	0 (0.0%)	17 (45.9%)
Registered permanent residence					
Urban	1 (25.0%)	14 (66.7%)	3 (27.3%)	0 (0.0%)	18 (48.6%)
Rural	3 (75.0%)	7 (33.3%)	8 (72.7%)	1 (100.0%)	19 (51.4%)
Commercial insurance					
Yes	4 (100.0%)	16 (76.2%)	10 (90.9%)	0 (0.0%)	30 (81.1%)
No	0 (0.0%)	5 (23.8%)	1 (9.1%)	1 (100.0%)	7 (18.9%)
Inclusive commercial insurance					
Yes	4 (100.0%)	14 (66.7%)	10 (90.9%)	0 (0.0%)	28 (75.7%)
No	0 (0.0%)	7 (33.3%)	1 (9.1%)	1 (100.00%)	9 (24.3%)
Disability					
Yes	3 (75.0%)	20 (95.2%)	7 (63.6%)	0 (0.0%)	30 (81.1%)
No	1 (25.0%)	1 (4.8%)	4 (36.4%)	1 (100.0%)	7 (18.9%)
Disability level					
Grade 1 Disability	3 (100.0%)	12 (60.0%)	2 (28.6%)	0 (0.0%)	17 (56.7%)
Grade 2 Disability	0 (0.0%)	7 (35.0%)	2 (28.6%)	0 (0.0%)	9 (30.0%)
Grade 3 Disability	0 (0.0%)	0 (0.0%)	3 (42.8%)	0 (0.0%)	3 (10.0%)
Grade 4 Disability	0 (0.0%)	0 (0.0%)	0 (0.0%)	0 (0.0%)	0 (0.0%)
Grade 5 Disability	0 (0.0%)	1 (5.0%)	0 (0.0%)	0 (0.0%)	1 (3.2%)
Civil relief					
Yes	1 (25.0%)	8 (38.1%)	4 (36.4%)	1 (100.0%)	14 (37.8%)
No	3 (75.0%)	13 (61.9%)	7 (63.6%)	0 (0.0%)	23 (62.1%)
Social assistance					
Yes	2 (50.0%)	9 (42.9%)	2 (18.1%)	0 (0.0%)	13 (35.1%)
No	2 (50.0%)	12 (57.1%)	9 (81.9%)	1 (100.0%)	24 (64.9%)
Current disease state					
Age of symptom onset (mean (SD)), years	0.3 (0.2)	0.9 (0.4)	3.2 (3.7)	19.0 (0.0)	2.0 (3.7)
Age at diagnosis (mean (SD)), years	0.6 (0.2)	1.8(1.3)	6.7 (8.5)	20.0 (0.0)	3.6 (5.9)
Scoliosis					
Yes	1 (25.0%)	19 (90.5%)	6 (54.5%)	0 (0.0%)	26 (70.3%)
No	3 (75.0%)	2 (9.5%)	5 (45.5%)	1 (100.0%)	11 (29.7%)
Number of hospitals visited before diagnosis	1.8 (1–2)	3.9 (1–13)	2.1 (0–10)	1.0 (1–1)	4.0 (1–13)
Caregiver and family					
Household size, n	3.3 (2–4)	4.8 (2–7)	4.1 (2–5)	3.0 (3–3)	4.4 (2–7)
Marital status					
Married	2 (50.0%)	21 (100.0%)	9 (81.8%)	1 (100.0%)	33 (89.2%)
Divorced	2 (50.0%)	0 (0.0%)	2 (18.1%)	0 (0.0%)	4 (10.8%)
Number of caregivers					
0	0 (0.0%)	0 (0.0%)	0 (0.0%)	1 (100.0%)	1 (2.7%)
1	4 (100.0%)	8 (38.1%)	4 (36.4%)	0 (0.0%)	16 (43.2%)
2	0 (0.0%)	6 (28.6%)	6 (54.5%)	0 (0.0%)	12 (32.4%)
3	0 (0.0%)	3 (14.3%)	0 (0.0%)	0 (0.0%)	3 (8.1%)
4	0 (0.0%)	4 (19.0%)	1 (9.1%)	0 (0.0%)	5 (13.5%)
Primary caregiver’s ^1^ (mean (SD)), y	36.3 (5.0)	40.1 (5.8)	36.5 (5.1)	25.0 (0.0)	38.1 (6.0)
Primary caregiver’s sex, n					
Male	0 (0.0%)	1 (4.8%)	2 (18.2%)	1 (100.0%)	4 (10.8%)
Female	4 (100.0%)	20 (95.2%)	9 (81.8%)	0 (0.0%)	33 (89.2%)
Primary caregiver’s employment status					
Employed full-time	0 (0.0%)	4 (19.0%)	1 (9.1%)	0 (0.0%)	5 (13.5%)
Employed part-time	0 (0.0%)	1 (4.8%)	3 (27.3%)	1 (100.0%)	5 (13.5%)
Unemployed	4 (100.0%)	16 (76.2%)	7 (63.6%)	0 (0.0%)	27 (73.0%)
Duration of caregiving for the SMA child per day, h	24.0 (24.0–24.0)	20.8 (5.5–24.0)	15.0 (3.0–24.0)	0.0 (0.0–0.0)	19.0 (3.0–24.0)
Family income, n					
RMB 10,000 to 60,000	3 (75.0%)	13 (61.9%)	6 (54.5%)	1 (100.0%)	23 (62.2%)
RMB 70,000 to 90,000	1 (25.0%)	4 (19.0%)	4 (36.4%)	0 (0.0%)	9 (24.3%)
RMB 100,000 to 130,000	0 (0.0%)	3 (14.3%)	1 (9.1%)	0 (0.0%)	4 (10.8%)
RMB 140,000 to 170,000	0 (0.0%)	0 (0.0%)	0 (0.0%)	0 (0.0%)	0 (0.0%)
RMB 180,000 to 210,000	0 (0.0%)	0 (0.0%)	0 (0.0%)	0 (0.0%)	0 (0.0%)
More than RMB 220,000	0 (0.0%)	1 (0.0%)	0 (0.0%)	0 (0.0%)	1 (2.7%)
Father education					
Junior high school or below	1 (25.0%)	5 (23.8%)	5 (45.5%)	0 (0.0%)	11 (29.7%)
Senior high school	2 (50.0%)	4 (19.0%)	2 (18.2%)	1 (100.0%)	9 (24.3%)
College/university or above	1 (25.0%)	12 (57.1%)	4 (36.4%)	0 (0.0%)	17 (45.9%)
Mother education					
Junior high school or below	1 (25.0%)	5 (23.8%)	6 (54.5%)	0 (0.0%)	12 (32.4%)
Senior high school	0 (0.0%)	5 (23.8%)	1 (9.1%)	1 (100.0%)	7 (18.9%)
College/university or above	3 (75.0%)	11 (52.4%)	4 (36.4%)	0 (0.0%)	18 (48.6%)

^1^ A type IV patient does not require caregivers other than himself; in the statistics, we list him as the primary caregiver.

**Table 2:** The basic situation of the interviewed caregiver’s DASS-21 scale test.

Variable	Frequency	Proportion (%)	DASS-21 Results		
			Stress	Anxiety	Depression
			Score x(s)	Score x(s)	Score x(s)
Relationship with patient					
Father	8	21.62	15.75 (9.22)	8.00 (8.94)	12.00 (10.31)
Mother	26	70.27	17.60 (9.18)	11.20 (6.43)	11.20 (6.43)
The patient him/herself	3	8.11	18.67 (8.32)	12.67 (5.03)	12.67 (5.03)
Whether primary caregiver ^1^					
Yes	30	81.08	17.40 (8.84)	10.33 (7.13)	15.07 (8.55)
No	7	18.92	16.80 (11.37)	12.00 (6.93)	12.00 (6.16)
Whether full-time caregiver					
Yes	28	72.97	18.37 (9.27)	11.85 (6.77)	16.59 (7.94)
No	9	27.03	14.00 (7.28)	6.89 (6.33)	8.00 (5.20)
Household registration					
Urban household registration	18	48.65	17.33 (8.60)	11.33 (5.90)	14.33 (7.36)
Rural household registration	19	51.35	17.22 (9.48)	9.89 (7.93)	14.55 (9.17)
Family income					
RMB 10,000 to 60,000	23	62.16	19.22 (8.20)	12.17 (6.03)	16.52 (7.29)
RMB 70,000 to 90,000	9	24.32	15.56 (10.09)	9.11 (8.25)	13.11 (9.23)
RMB 100,000 to 130,000	4	10.81	12.67 (6.43)	6.67 (7.02)	5.33 (5.77)
RMB 140,000 to 170,000	0	0.00	/	/	/
RMB 180,000 to 210,000	0	0.00	/	/	/
More than RMB 220,000	1	2.70	2.00 (0.00)	0.00 (0.00)	6.00 (0.00)
Type of SMA					
I	4	10.81	12.50 (5.74)	7.00 (3.46)	13.50 (3.00)
II	21	56.76	16.60 (9.27)	10.30 (7.35)	14.70 (8.57)
III	11	29.73	20.36 (9.20)	12.36 (7.26)	14.91 (9.40)
IV	1	2.70	16.00 (0.00)	12.00 (0.00)	8.00 (0.00)
The therapeutic condition of the patient					
Never received treatment	6	16.22	17.00 (9.90)	7.00 (7.07)	14.00 (5.66)
Currently not receiving treatment	1	2.70	16.00 (0.00)	11.00 (0.00)	16.00 (0.00)
Receiving treatment	30	81.08	17.37 (8.91)	10.80 (7.00)	14.33 (8.66)

^1^ A type IV patient does not require caregivers other than himself; in the statistics, we list him as his primary caregiver and full-time caregiver.

**Table 3:** Self-assessment of medication and rehabilitation satisfaction among SMA patients’ families.

	Self-Assessment of Medication Satisfaction					Self-Assessment of Rehabilitation Satisfaction				
	Very Dissatisfied	Dissatisfied	Neutral	Satisfied	Very Satisfied	Very Dissatisfied	Dissatisfied	Neutral	Satisfied	Very Satisfied
Type of SMA										
I	0	1	2	1	0	0	1	1	1	0
II	0	6	8	1	0	0	4	9	5	0
III	0	0	6	4	1	0	1	6	2	1
IV	0	1	0	0	0	0	0	1	0	0
Scoliosis										
No	0	2	5	2	0	0	0	5	4	0
Yes	0	6	11	4	1	0	5	13	4	1
Gender										
Male	0	6	8	3	0	0	0	8	4	1
Female	0	1	9	3	1	0	5	10	4	0

Note: The self-assessment satisfaction questionnaire included only patients receiving medication (n = 31) and those receiving rehabilitation (n = 32).

## Data Availability

The data presented in this study are not available due to privacy.
